# Sampling event dataset on five-year observations of macrofungi fruit bodies in raised bogs, Western Siberia, Russia

**DOI:** 10.3897/BDJ.7.e35674

**Published:** 2019-07-30

**Authors:** Nina Filippova, Elena Lapshina

**Affiliations:** 1 Yugra State University, Khanty-Mansiysk, Russia Yugra State University Khanty-Mansiysk Russia

**Keywords:** Macrofungi, plot-based protocol, peatlands, phenology, permanent monitoring

## Abstract

**Background:**

The data paper includes the results of a long-term monitoring programme for macrofungi fruiting using permanent plots located in the raised bog ecosystem in central part of Western Siberia (nearby Khanty-Mansiysk), Russia. The goal of the project was to describe the quantitative and qualitative structure and spatial variation of the community of macromycetes, to follow its dynamics seasonally and inter-annually and also elucidate the relationship between the fruiting and climate variables. A total of 263 circular 5 m^2^ subplots (for a total area of 1,315 m^2^) were inspected weekly during vegetation seasons 2014-2018 and carpophores of different fungal taxa were counted. The resulting sampling-event dataset includes 16,569 of plot-based observations (= sampling events) with corresponding 6,011 occurrence records of macromycetes identified to species or genus level. In total, 69 species were revealed during the study. About 80% of plot-based observations contain zero records and mark absence of visible fruiting bodies in a certain plot and time.

**New information:**

This is the first sampling-event dataset on plot-based observations of macrofungi published in GBIF and the first long-term series of macrofungi monitoring in a raised bog ecosystem accomplished in Western Siberia. The aim of the data paper publication was to provide the description and the link to the published data in the format of a peer-reviewed journal paper and to provide recognition for the effort by means of a scholarly article (based on Data paper definition published at https://www.gbif.org/en/data-papers).

## Introduction

Ombrotrophic raised peatlands (bogs) are unique ecosystem types with special flora and microbial composition and which store large amounts of terrestrial carbon, yet these ecosystems are vulnerable and lost in many countries due to peat excavation and drainage (Vitt and Wieder 2006). The peatland mycobiota is relatively poor by species number, but rich in habitat-specific species playing sufficient role in decomposition of the upper aerated peat layer. Macrofungi are important components of the peatland mycobiota, playing a key role of saprotrophs and forming mycorrhizal relationships with plants (Thormann 2006). The classical methods of direct observation of fruiting structures using permanent plots is essential to comprehend the spatial and temporal scales of the community of macrofungi. The results of long-term observations could reveal several important community characteristics, like species composition and quantitative and qualitative structure, spatial heterogeneity, phenology, inter- and intra-annual community dynamics and ecology of different species.

The protocols for sampling macrofungi using fixed-sized plots have begun to converge in recent years (O'Dell et al. 2004), thus making possible the comparison of different project results and allowing extrapolation of data. On the other hand, development of integrated biodiversity information facilities has opened the possibility of standardised data storage and open access to the accumulated quantitative data, together with the habitat characteristcs where they were collected. The sampling-event dataset type of GBIF allows publication of data on quantitative community assessments, like vegetation transects, bird censuses and freshwater or marine sampling. The assessments of macrofungi community are still absent or rare in GBIF, despite the importance of such data for ecological monitoring and rare species assessment.

Following the standard plot-based protocol (Mueller et al. 2004 - original publication, Mueller et al. 2019- publication of the same protocols at protocols.io) for sampling macrofungi, we considered the sampling event being a single subplot observation on a particular date where all epigeous carpophores (fruit bodies) of each species inside the subplot were indentified and counted. The subplots had the unique numbers (parentEventID) making possible the enumeration of the accumulated number of sporocarps on each subplot after a number of visits, as well as the study of spatial heterogeneity in relation to habitats. Every subsequent visit of a subplot was uniquely identified by the eventID number (and a date), thus allowing the study of inter- and intra-annual dynamics. The resulting dataset includes two tables: the event table with a description of events (micro-plots and dates of observations) and the occurrence table with lists of counted carpophores on each event.

Thereby, the sampling-event dataset of macrofungi observations represents data on fungal community quantitative and spatial structure and its dynamics. These data could be important for studying the whole mycobiota or ecology of particular species, making decisions on specificity of habitats of raised bogs, ecological niches modelling and influence of climatic parameters on macrofungi fruiting.

## Project description

### Title

Plot-based observations of macrofungi in major ecosystem types of taiga zone, Western Siberia

### Personnel

Nina Filippova, Elena Lapshina

### Study area description

The area of study is located in the middle taiga zone of Western Siberia. For the purpose of permanent monitoring of fungal communities of local ecosystems, two sites were chosen at about 20 km SW and E from the Khanty-Mansiysk town nearby two field stations of Yugra State University.

### Design description

The recommended protocols for sampling macrofungi were followed during the project (O'Dell et al. 2004, Mueller et al. 2019). A series of subplots was established in a raised bog (totally 263) (corresponding to two "virtual plots" with two different vegetation types). Another ten plots were established in different forest types following after-logging and paludification successions (in total, 300 subplots). These plot-based observations were also supplemented by opportunistic routs to record rare species and habitats. Due to variations in fruiting in relation to weather conditions and internal fruiting dynamics, the plot-based monitoring should be continued for at least 10 years and this project will try to follow these goals; the datasets will be updated after new data have been collected.

### Funding

The project is partially funded by the Yugra State University grant #13-01-20/39. Permanent monitoring at the Mukhrino Field Station is also supported by INTERACT – International Network for Terrestrial Research and Monitoring in the Arctic (https://eu-interact.org).

## Sampling methods

### Study extent

**Figure 1.**
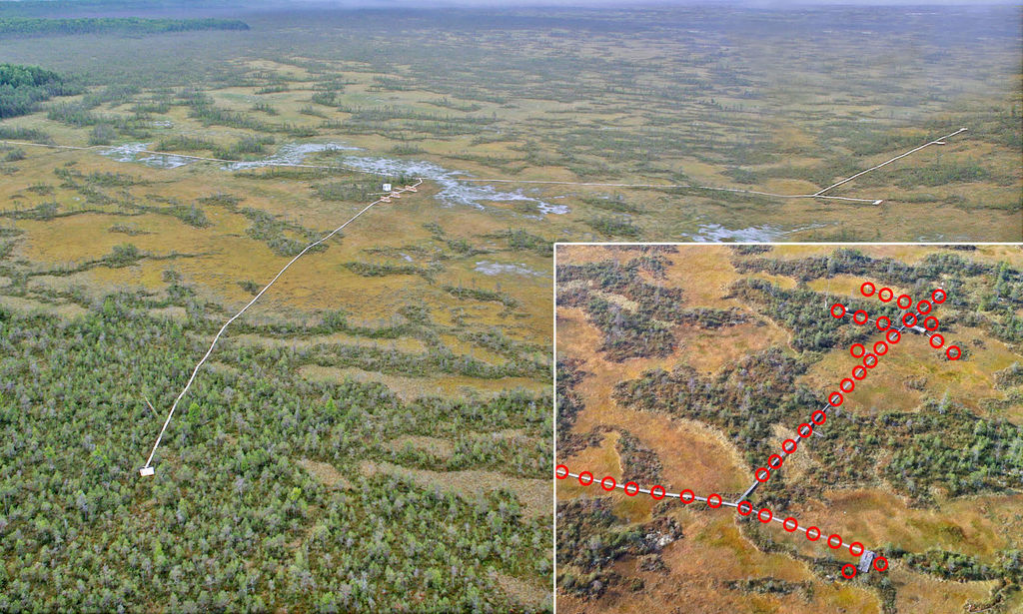
General view of the Mukhrino Fields Station infrastructure and position of subplots for observation of macromycetes located along the walking board (in the insert).

The long-term monitoring plots were located along a walking board of the research polygon of Mukhrino Field Station (Fig. 1), thereby eliminating any future impacts to the bog as a result of ongoing research programmes. A total of 263 circular x 5 m^2^ (for a total area of 1,315 m^2^) long-term monitoring subplots were established in May 2014. This total area is nearly evenly divided between the two major plant communities (two "virtual plots"): Pine-dwarfshrubs-Sphagnum dominated bogs (dominated by *Pinus sylvestris*, *Chamaedaphne calyculata*, *Ledum palustre*, *Rubus chamaemorus*, *Sphagnum fuscum*) and Graminoid-Sphagnum lawns (dominated by *Scheuchzeria palustris*, *Carex limosa*, *Eriophorum russeolum*, *Oxycoccus palustris*, *Sphagnum balticum*) and incorporates plant community variation. An area of about 700 m^2^ is sufficiently large to reveal the fungal community diversity in raised bogs, based on species accumulation curves constructed from these data.

### Sampling description

The recommended protocols for sampling macrofungi were followed in general terms (O'Dell et al. 2004). Four steps of the protocol: selection of observation site, transects and subplots installation, carpophore counting and collecting specimens are described in detail in a published protocol by Mueller et al. (2019).

### Quality control

About 1,000 dry specimens were collected in line with the project. The collection database is available at: (https://fungariumysu.org/fungarium-ysu-database; https://www.gbif.org/dataset/d922b606-6c94-4d51-9277-36c9b03872a7). All identifications were made by the first author and some collections were sent to experts in a particular taxonomic group for proper naming. Future thorough taxonomic work is also necessary for some taxonomically difficult genera like *Cortinarius*, *Russula* and *Galerina*.

### Step description

**Figure 2.**
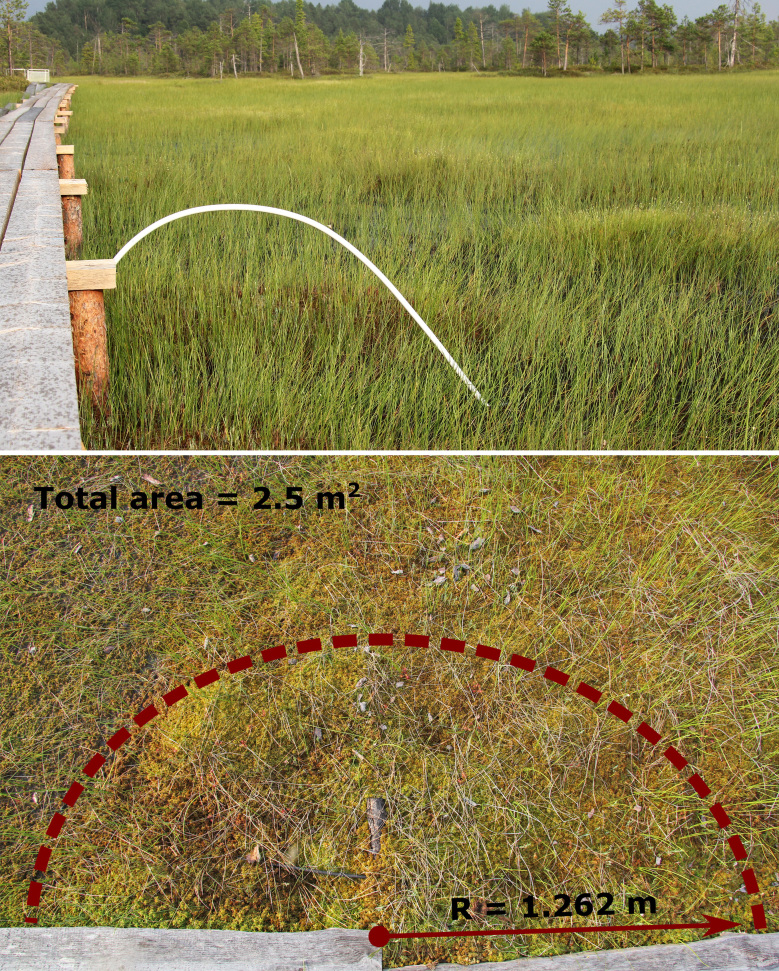
The example of examination of a subplot located in Graminoid-Sphagnum lawn community using a bent bow to draw the outlines (total area of a circular subplot = 5 m^2^)

In order to preserve the peatland surface, the long-term monitoring subplots were located 5 m apart in a straight line along the walking boards throughout their length. The plots were chosen by ensuring that they fell only into a single plant community. Centres of each subplot were marked with a metal label on the walking board side and a bent bow was used to draw the outlines of a subplot during its examination (Fig. 2).

A total of 263 circular 5 m^2^ (for a total area of 1,315 m^2^) long-term monitoring subplots were established in May 2014. The subplots were inspected weekly from May to September in 2014-2018 (except for August 2017 when observations were interrupted and some occasional gaps in observations which were interpolated during the following quantitative analyses). All carpophores of each species were counted and collected for subsequent examination. Enumerated carpophores were carefully removed from the subplots, with the exception of Red Listed taxa (*Ascocoryne turficola*, *Entoloma fuscomarginatum*, *Geoglossum sphagnophilum*, *Hygrocybe cinerella*, *Mycena concolor*, *Omphaliaster borealis*, *Psilocybe turficola*), whose carpophores remained untouched.

Climatic data (precipitation, air temperature, soil profile temperature) were collected from a micro-climate monitoring station established nearby the plots (https://mukhrinostation.com/research/weather-station/). The description of vegetation was made in each subplot using the general *relevé* approach.

The common and easily recognisable species were identified in the field. The detailed identification of doubtful species was done in the laboratory. Most of the specimens were identified using Funga Nordica keys (Knudsen and Vesterholt 2008) and some additional monographs on particular taxa were used when necessary. The old fruiting bodies which were difficult to assign to a particular species were left with the genus level identification (about 100 of this kind of records in the database).

The collections were processed as described in Lodge et al. (2004). Fresh fruiting bodies were wrapped in aluminium foil and carried to the laboratory to be processed on the day of collection. The processing of specimens included:

1. photographing on a photo-studio table;
2. description of vital characters;
3. preliminary microscopy and identification;
4. filling the data in the database;
5. labelling;
6. drying at 50°C to store in the Fungarium of Yugra State University;

The detailed identification was done during the winter following the collection season. Dry specimens were rehydrated in tap water or KOH (10%); dyes and other chemicals (Congo Red, Melzer's reagent, ammonia) were applied when necessary. A Zeiss Axiostar microscope with Achromat 5/0.12, 10/0.25, 40/0.65 (dry) and 100/1.25 (oil immersion) objectives was used for microscopical examination.

## Geographic coverage

### Description

The studied area is located in the middle taiga zone of Western Siberia. Mukhrino Field Station of Yugra State University was established 30 km SW from Khanty-Mansiysk, nearby the Mukhrina River (left inflow of the Irtysh River). The research polygon of the station, located in a bog, has the infrastructure of walking boards along which the monitoring programme was established. The subplots for long-term monitoring of macrofungi were located along the boardwalk line within the radius of about 500 m from the central coordinate of the infrastructure (60.89188N, 68.68233E).

### Coordinates

60.889 and 60.896 Latitude; 68.670 and 68.692 Longitude.

## Taxonomic coverage

### Description

Terrestrial macrofungi (larger fungi) were studied during the survey. The group was defined as the macrofungi which are confined to terrestrial habitat as opposed to wood-inhabiting species representing another prominent community in boreal forests. However, these groups partially overlap and we recorded species growing on mossy old trunks or buried wood within the plots. Our study included the following groups in the analysis: Discomycetes, Agaricoid, Boletoid, Aphyllophoroid fungi (we omitted brackets, crusts and jellies but included clubs and coral fungi) and some other groups in minority. The taxonomic coverage includes representatives of two divisions (Ascomycota - 4 species, Basidiomycota - 65), three classes (Agaricomycetes - 65, Leotiomycetes - 2, Pezizomycetes - 2) and 22 families (Amanitaceae - 1, Auriscalpiaceae - 1, Boletaceae - 1, Clavariaceae - 1, Cortinariaceae - 16, Entolomataceae - 2, Helotiaceae - 1, Hydnangiaceae - 1, Hygrophoraceae - 4, Hymenogastraceae - 12, Inc. sed. - 1, Inocybaceae - 1, lyophyllaceae - 1, Mycenaceae - 5, Omphalotaceae - 3, Physalacriaceae - 1, Russulaceae - 5, Sarcosomataceae - 2, Sclerotiniaceae - 1, Strophariaceae - 3, Suillaceae - 2, Tricholomataceae - 4 species).

### Taxa included

**Table:** 

Rank	Scientific Name	Common Name
kingdom	Fungi	Mushrooms

## Traits coverage

### Data coverage of traits

PLEASE FILL IN TRAIT INFORMATION HERE

## Temporal coverage

### Notes

From 2014 through 2018, the subplots were inspected weekly from May to September

## Collection data

### Collection name

Fungarium, part of the Yugra State University Biological Collection

### Collection identifier

YSU-F

### Parent collection identifier

YSU (http://sweetgum.nybg.org/science/ih/herbarium-details/?irn=244549)

### Specimen preservation method

dried

## Usage rights

### Use license

Creative Commons Public Domain Waiver (CC-Zero)

## Data resources

### Data package title

Plot-based observations of macrofungi in raised bogs in Western Siberia

### Resource link

http://gbif.ru:8080/ipt/archive.do?r=bogfunplots

### Alternative identifiers

https://doi.org/10.15468/e9g5ri

### Number of data sets

1

### Data set 1.

#### Data set name

Plot-based observations of macrofungi in raised bogs in Western Siberia

#### Data format

Darwin Core

#### Number of columns

27

#### Character set

UTF-8

#### Download URL

https://www.gbif.org/dataset/acd76923-54da-4799-b4b0-cfe585c2c0b8

#### Description

The dataset includes two related tables of Darwin Core format, the basic *Event* table and the related *Occurrence* table (Filippova 2019). The *Event* table includes 20 fields and 16,569 records. The fields include description of habitat, geography, date and subplot size. All subplots observations were registered including those with absence records of fungi, 163 subplots by 63 visits totalling in 16,569 records. The *Occurrence* table includes 7 fields and 6,011 records. The fields include the scientific name and carpophore counts within each subplot on a particular date. The two tables are related by the eventID field. The absence of occurrence records corresponding to the record in the Event table marks absence of fungi within a subplot on a particular date.

**Data set 1.** 

Column label	Column description
eventDate (Event table)	Date of subplots examination
parentEventID (Event table)	A unique number of a subplot
eventID (Event table)	Unique identifier of a particular visit of each subplot
habitat (Event table)	Vegetation cover
decimalLatitude (Event table)	Geographic latitude
decimalLongitude (Event table)	Geographic longitude
geodeticDatum (Event table)	Geodetic datum
coordinateUncertaintyInMeters (Event table)	Coordinate uncertainty in metres
coordinatePrecision (Event table)	Coordinate precision
minimumElevationInMetres (Event table)	Minimum elevation
maximumElevationInMetres (Event table)	Maximum elevation
sampleSizeValue (Event table)	The size of a subplot
sampleSizeUnit (Event table)	Size unit
sampingProtocol (Event table)	Sampling protocol
country (Event table)	Country
countryCode (Event table)	Country code
stateProvince (Event table)	Province
municipality (Event table)	The nearest town
locality (Event table)	Locality
type (Event table)	The name of the resource
eventID (Occurrence table)	Unique identifier of a particular visit of each subplot
occurrenceID (Occurrence table)	Unique identifier of a particular observations of each species within a subplot
basisOfRecord (Occurrence table)	Basis of record (human observation)
Kingdom (Occurrence table)	Kingdom
scientificName (Occurrence table)	Scientific name
organismQuantity (Occurrence table)	Number of carpophores
organismQuantityType (Occurrence table)	Quantity type (number of fruitbodies)
